# Satellite Cells: Regenerative Mechanisms and Applicability in Muscular Dystrophy

**DOI:** 10.1155/2015/487467

**Published:** 2015-02-11

**Authors:** Gustavo Torres de Souza, Rafaella de Souza Salomão Zanette, Danielle Luciana Aurora Soares do Amaral, Francisco Carlos da Guia, Claudinéia Pereira Maranduba, Camila Maurmann de Souza, Ernesto da Silveira Goulart Guimarães, João Vitor Paes Rettore, Natana Chaves Rabelo, Antônio Márcio Resende do Carmo, Fernando de Sá Silva, Carlos Magno da Costa Maranduba

**Affiliations:** ^1^Laboratory of Human Genetics and Cell Therapy, Biology Department, Biological Sciences Institute, Federal University of Juiz de Fora, 36036-900 Juiz de Fora, MG, Brazil; ^2^Department of Endodontology, Dentistry School, Federal University of Juiz de Fora, 36036-900 Juiz de Fora, MG, Brazil

## Abstract

The satellite cells are long regarded as heterogeneous cell population, which is intimately linked to the processes of muscular recovery. The heterogeneous cell population may be classified by specific markers. In spite of the significant amount of variation amongst the satellite cell populations, it seems that their activity is tightly bound to the paired box 7 transcription factor expression, which is, therefore, used as a canonical marker for these cells. Muscular dystrophic diseases, such as Duchenne muscular dystrophy, elicit severe tissue injuries leading those patients to display a very specific pattern of muscular recovery abnormalities. There have been works on the application of precursors cells as a therapeutic alternative for Duchenne muscular dystrophy and initial attempts have proven the cells inefficient; however later endeavours have proposed solutions for the experiments improving significantly the results. The presence of a range of satellite cells populations indicates the existence of specific cells with enhanced capability of muscular recovery in afflicted muscles.

## 1. Introduction

In the scientific literature, muscle tissue is often related to the ability of considerably fast recovery from injuries, as well as to the plasticity due to adaptation to stress provoked by strenuous stimuli of the muscular fibers in various manners, such as exercising [1, 2]. The recovery of the cytoarchitecture of the muscular tissue has been reported to happen within the considerably short period of two weeks [1]. The process of restoration of the conditions of the tissue is subject to a series of molecular events and cell signalization. Nevertheless, the regeneration capacity of muscle tissue is limited to a certain extent; and the fact that skeletal muscle cells fully differentiate into myofibers which are known to be in mitotic arrest settled due to the cell cycle inhibitor effect of the retinoblastoma protein (pRB) [3–5] would suggest the muscle tissue to lack plasticity and ability to recover from injuries. However, the discovery of the presence of resident progenitors or adult stem cells [ASCs] surrounding the myofibrils could successfully explain the dynamics of this tissue [6, 7]. These cells have been generally related to as satellite cells (SCs) due to the very location they had in relation to the myofibrils; howbeit, the general application of this term does not refer formally to a specific ASCs population [6, 8]. In adult life, the SCs are displayed in a quiescent state in skeletal muscles, surrounding the myofibrils and placed adjacently to the basal lamina. Upon stimuli caused by tissue injury those cells regain activity and fuse to the myofibrils recovering them or between SCs in order to form new fully differentiated skeletal muscle cells. In addition, the SCs undergo self-renewing mitosis maintaining their number in the tissue after the recovery [1, 9–13].

Considering the importance of the SCs in the recovery of the muscular tissue, it allows us to predict the relevance of those cells and other ASCs in proposing cell-based therapies for myopathies as well as in the understanding of their pathogenesis [9, 10]. Among the diseases afflicting the skeletal muscle tissue is Duchenne muscular dystrophy (DMD), which is caused by frame shift mutations in the dystrophin gene located in the locus Xp21. The mutated protein may lead to severe muscle cellular damage due to alterations in the cytoskeleton, characterizing this disease as a congenital myopathy; however it must be considered that the severity of the phenotype presented by the patient is connected to the mutation site, giving rise to a variety of conditions in response to this mutation [14–16]. DMD is a disorder with early onset, in which the affected individual presents weakness and difficulties in controlling the muscular movements in the childhood and culminates with severe conditions involving cardiomyopathy and respiratory complications leading to death around the third decade of life [14–17]. The lack of efficacious established treatment turns necessary the evaluation of different approaches to attempt treating the affected patients. Thus, the understanding of the mechanisms of recovery performed by SCs gains great importance in attempting to promote possible new cell-based therapies for this disease. The present review aims at compiling relating the molecular mechanisms underlying the muscular recovery by the SCs, which may be involved in the process in order to associate them with the pathogenesis and possible treatment perspectives of DMD.

## 2. Myogenic Stem Cell Populations the Muscular Tissue

The distinct capacity for muscle regeneration has been long thought to have the SCs which were the sole contributors; however, the involvement of other ASCs populations has been later determined [18–21], as well as the heterogeneity of the very SCs [8]. The biological events involved in the control of all stem cells which have a role in the process of recovery of the muscle tissue are tightly controlled by molecular mechanisms, which will later be discussed in this review [22]. There seems to be varied cell populations within the muscle to which the myogenic capacity may be attributed [8]. Those are often referred to in the literature as muscle-ASCs; nonetheless this review will focus on the heterogeneous population defined as SCs, composed by cell types with a more stem cell-like profile and more tissue-committed cells [8].

### 2.1. Satellite Cells (SCs)

In 1961, electron microscopy allowed Mauro to first observe the presence of the SCs, mononucleated cells localized in the periphery of the skeletal myofibers of frogs [6]. The existence of this cell type was later discovered in other animals, including humans, and their involvement in tissue regeneration was determined [23–25]. The very location of these cells, juxtaposed to the myofibrils, would suggest their involvement in muscle regeneration. This hypothesis began to be proved by the experiments of Snow in 1977, in which H^3^ marked thymidine allowed inferring valuable information on how these cells would contribute to the muscular regeneration. These experiments indicated that the SCs remain quiescent in the mature muscle and regain mitotic activity following tissue injuries; in addition, differentiation to myoblasts was stimulated, which then contributed to the nuclei of the myofibrils resulting in the recovery of the injury [26]. In 1975 two* in vitro* experiments had already given important insights into how muscular recovery happens [27, 28]. On both studies cell culture techniques were used to observe how regeneration occurred on single myofibers. The mononucleated cells, presumed to be the SCs, were found to soon enlarge and begin proliferation and finally fuse to the multinucleated myofibrils resulting in their recovery [27, 28].

Although the SCs were known to participate in muscle recovery, it was still unclear whether they were resident ASCs, progenitor cells, or dedifferentiated cells. Later experiments showed that SCs could both successfully differentiate into myoblasts and recover injured myofibrils and undergo self-renewal. Those characteristics allowed them to be classified as ASCs. In 2005, Collins et al. performed experiments to evaluate the self-renewal and differentiation capacity. Single myofibers were transplanted into previously radiated muscle, demonstrating that the limited number of SCs in the transplanted fiber was sufficient to generate a significant amount of multinucleated myofibrils as well as to recover the tissue after experimental injury had been performed in it [29]. Later, the findings of Kuang et al. corroborated to the previous results, by concluding that the SCs would perform self-renewal by asymmetric cell divisions. In addition, these experiments also allowed the important observation that the SCs were not composed of a single population of ASCs, rather populations of ASCs and committed progenitor cells [30].

SCs were previously simplistically regarded as cells of a single population with conserved markers throughout distinct muscles of the body [27]. However, difference in embryonary origin of some muscles has appeared as an important indicator that the SCs populations were as well distinct, once their origin is the same as the muscle where they reside. Evidences from recent studies concerning the embryogenesis have shown the skeletal muscles of the trunk and limbs share a common origin, whereas the muscles covering the cranium are originated in other embryonary structures of the cranial mesoderm [31–35]. Advances regarding molecular biology in the last decades allowed observing the existence of different SCs populations inside a certain muscle as well as distinct molecular signatures specifically connected to the anatomic position of the muscle [8].

There are significant dissimilarities among the composing muscles of the body in what concerns their physiological aspects. The cited embryonary distinction of the muscles implicates differences in their rate of regeneration [36] as well as in the propensity to express the phenotype of inherited disorders [37]. As an extension, the specific populations of SCs in the different muscles may be directly linked to the muscular disorders such as DMD and, hence, bear relevance while studying the molecular basis and possible treatments for these diseases [36]. For that matter, the study of the molecular markers in SCs populations gains noteworthiness, not only as a basic research field but also for their clinical correlation to muscular genetic disorders.

Substantial work has been performed in determining the molecular biomarkers which appear in almost all the quiescent SCs and, therefore, determine their identity. The most widely documented SCs-related molecule is the paired box homeotic protein (Pax) 7, which is a transcription factor, related to the very embryonary development of those cells and is constitutively expressed in both quiescent and proliferating SCs [38]. In spite of the high variability of SCs populations, there exist determining markers, which appear in most of those cells and, therefore, may be used to identify those cells. In order to review the molecular profile of most of the adult skeletal muscle SCs, the already identified markers, as well as Pax7, are summarized in Table 1.

Despite the fact that the markers displayed in Table 1 can widely characterize SCs, there has been ongoing research on elucidating molecules that enable the distinction between different populations of those cells [8]. In addition to the difference in the molecular profile of those cells, the different muscles also exhibit a different density of this sort of cell [29, 39–41]. Embryological origin seems to be tightly related to the expression of some proteins in SCs; the differential expression of Pax3 and Pax7 transcription factors have been evaluated by several studies during the myogenesis, allowing the conclusion that this expression is related to the various myogenic states of the somite as well as to the embryo region from which those cells derive ([42], for review, see [8]). In 2005, Kassar-Duchossoy et al. successfully demonstrated the Pax3+ and Pax7+ to be responsible for giving rise to the muscle progenitor cells [43]. These results were largely corroborated by other studies, which also showed the Pax-dependency on the myogenetic process, in addition to the differential expression of those markers in the various stages of embryogenesis [38, 44–46]. Later, the roles of Mrf4 and Myf5 in the muscle development have been elucidated and the cells expressing those genes during embryonic period have been shown to be responsible for giving rise to a significant part of the SCs [47, 48].

In addition to the varied molecular profile of the cells giving rise to the SCs, these progenitor cells also display difference in the markers expressed in distinct muscles or distinct proliferation status of the cell, which may be related, to a certain extent, to their physiological function in that anatomical location or within the same muscle. In 2000, Beauchamp et al. have found that CD34 and Myf5 are expressed in SCs in different stages of muscle development. However, results showed the existence of a CD34−, Myf5−, and M-cadherin− population, which was then speculated to be representative of a less differentiated population [49]. Later, the expression of CD34 was compared to the regenerative capacity of the myogenic cells [50]; considerations made were that the CD34+ cells would more promptly fuse and regenerate muscular injury, in a manner that their transplantation in dystrophic host mice yielded a more extensive dystrophin restoration [50]. Kuang et al. (2007) have perceived the heterogeneity of the SCs population by evaluating the expression of Myf5. It has been found that approximately 10% of the Pax7+ cells in the sublaminar area did not express Myf5; however, the Pax7+ and Myf5− cells give rise to both Myf5-expressing and Myf5-nonexpressing cells through asymmetrical mitosis. When the SCs undergoing apical-basal-oriented mitosis, the apical cells were Myf5+, whereas the basal cells did not [30], thus giving rise to two different populations. The Myf5− population was related to self-renewal of that SCs niche, while the Myf5+ cells were concluded to be more committed [51].

SCs from muscles in different anatomical sites with different embryonic origin display substantially different features, which include the presence of varied markers, both in embryonary and adult life [52]. The differences in those SCs implicate altered behaviour during muscular recovery and, thus, may be related to the ability to recover from injuries inflicted by myopathies as well as from the propensity to the affection by this class of diseases [52]. Although it is predictable that the SCs resident in each muscle display a slightly different pattern of biomarkers, only some anatomical sites have been regarded in the scientific literature so far. The SCs in the head (concerning their similar embryonic origin) have been shown to significantly differ from those in other muscles, that the SCs from the head show cardiogenic potential being remarked, which did not appear in the other SCs studied [34]. Sambasivan et al. (2009) have shown the differential expression of some transcription factors and other regulatory proteins to be involved in the fate of SCs in different body locations, also allowing these proteins to be used as possibly more specific markers to the progenitor cells of some muscles. It has been noted that the SCs of some specific muscles show considerably different patterns of expression of their markers as well as distinct relations of those molecules and the activation of those cells. In extraocular muscles, either Myf5 or Mrf4 is indispensable for the development of SCs; in the pharnyx, Mrf4 has been shown to be necessary for the fate of SCs, being the transcription box factor 1 (Tbx1) also found to be expressed in this location [35]. Furthermore, later reports by Ono et al. (2010) demonstrated the difference in molecular profile between the extensor digitorum longus (EDL), masseter (MAS), soleus (SOL), and extensor carpi radialis (ECR) muscles (for review, see [39]).

The already cited experiments by Collins et al. also started to provide early insight into the heterogeneity in the functionality of SCs [29]. In these experiments, a single mice myofiber with its surrounding SCs was transplanted into the previously irradiated mice muscles. The results showed that the myofibers originated from the tibialis anterior (TA) could generate less new myofibers than those from the EDL or soleus [29]. These findings allow concluding the existence of different regenerative capacity intrinsic to the very SCs. Such difference may lead to important clinical correlations regarding the recovery from myopathic injuries to be later discussed (for review of markers and adult and embryonic regions, see [8, 53]). These results have been corroborated by the experiments by Sacco et al. (2008), where single SCs cells were transplanted into radiation-ablated muscles showing that the Pax7+ cells were responsible for the regeneration in the recipient muscle [54].

SCs have been reported to diminish in number and myogenic capacity in a direct relation to the age of the individual. There is evidence for the reduction in number of those cells; nevertheless, the myogenic capacity does not seem to be affected, suggesting the necessity for further research on the remaining cell populations and underlying mechanisms of the myogenic capacity with the advance of the age [55–62].

The data reviewed above allows characterizing the SCs as important cell populations involved in muscle recovery. In spite of the largely debatable age-dependent SCs depletion and loss of myogenic capacity [55–62], those cells keep an important role in muscle physiology and, thus, have great potential to be explored in the possible cell-based therapies for severe myopathies, such as DMD, which will be later discussed.

## 3. Isolation and Culture of SCs

A significant portion of the experiments cited relies to some extent on cell culture techniques and isolation of SCs, which are relevant for both studying the very cell population and for allowing their subsequent transplantation. Considering the relevance of the procedures involved in cultivating SCs and the distinction upon comparison to the methods used for other cell types, it becomes important to present the accepted techniques for that purpose [53, 63, 64]. The adherent feature of the SCs plays a major role in the practical aspect of harvesting those cells for further isolation procedures, as later described, and all* in vitro* assays regarding these lineages.

In 2004, Fukada et al. have successfully isolated murine SCs by employing a novel monoclonal antibody. In this study, the antibody SM/C-2.6 could bind specifically to the SCs in muscle preparations. In addition, the SM/C-2.6+ cells isolate via fluorescence-activated cell sorting (FACS) which could be successfully differentiated into myoblasts and myotubes* in vitro*, under specific differentiation conditions.* In vivo* differentiation into myofibers has also been performed with cells isolated from GFP-expressing mice by using the same antibody. These results indicate the antibody developed by the group to be a useful novel tool for initiating culture procedures with SCs in order to enable further studies regarding those cells [63].

Direct isolation of SCs has also been successfully achieved through flow cytometry techniques. The cells were isolated from preparations of adult GFP-expressing mice diaphragm muscle. The fraction selected was of Pax3+ and the experiments performed allowed concluding that those cells are a homogenous population of SCs, also expressing Pax7+ and CD34+ after cell sorting [64].

Motohashi et al. have recently proposed a more recent isolation and culture protocol for the same purpose. The protocol involving magnetic-activated cell sorting (MACS) reduces the costs of the process in relation to FACS [53]. The protocol is summarized in Table 2.

## 4. Molecular Response Involved in Muscle Recovery by SCs

### 4.1. Earlier Experiments Regarding SCs Activation and Proliferation

Muscular recovery relies greatly on the activity of SCs, which involves, simplistically, these cells leaving their quiescent status in order to undergo mitosis and fuse to the injured myofibers, reestablishing their cytoarchitecture and functionality [65]. In order to fulfill their role, SCs must be able to respond to the various stimuli, which may afflict the muscles and require their restorative activity. In 1986, Bischoff provided the earliest insights into the mechanisms of this recovery. It was shown that the quiescent SCs would enter cell cycle after exposure crushed muscle in order to elicit the stimulation, thus allowing prediction of the existence of a certain mitogen in the muscle, which stimulated SCs activation [66]. In other experiments, the author showed that the unstimulated SCs displayed little proliferative capacity; however mitosis could be induced by the addition of chick embryo extract or fibroblast growth factor [67]. Subsequently, transforming growth factor-b (TGF-b), insulin-like growth factor I (IGF-I), and fibroblast growth factor (FGF) was discovered to be implicated in the cellular mechanisms for controlling both proliferation and differentiation of SCs. While TGF-b was found to depress the proliferation rate and inhibit differentiation, IGF-I has positive effect over both factors (TGF-b and FGF) and over FGF stimulated proliferation, inhibiting differentiation, corroborating to the findings by Bischoff [68–70]. Later studies evaluated the effect of TGF-b over the porcine SCs proliferation in the presence of other cell signalization factors. The various combinations between TGF-b and platelet-derived growth factor (PDGF), FGF, IGF-I, and epidermal growth factor (EGF), resulted in observing distinct roles for the TGF-b depending on the factor with which it interacts, thus showing the mitosis and differentiation regulatory system of SCs to be dependent on various factors rather than specifically on one molecule [71–75]. The findings thus far allowed identifying the existence of an intricate underlying pathway for controlling SCs proliferation and differentiation, in addition to the occurrence of a mitogen for those cells in the very muscle.

### 4.2. Events Related to SCs Self-Renewal and Activation

The advances in the molecular biology techniques allowed unraveling the intrinsic mechanisms involved in both the processes of self-renewal and activation followed fusion to the damaged myofiber. Self-renewal capacity is essential for those cells to maintain their needed number to perform their tissue regeneration role. As already stated, among the SCs populations, there may be identified two distinct populations being the Myf5− cells less committed to the myogenic lineage and more prone to self-renewal than the Myf5+ [51]. In addition to that single gene expression, there exist various factors which implicated the self-renewal potential. This capability is directly related to the maintenance of tissue regeneration, considering that depleting those cells would implicate a severe decrease in the ability to recover from injuries, in a manner that it is tightly bound to the dystrophies myogenesis, considering the occurrence of muscle injuries in an extended time in those conditions [14–16, 52]. Pax7 and Pax3 expression seem to have a more direct role on the self-renewing process. It has been shown to lie upstream as a transcription factor in the regulatory pathways of the myogenic determinant genes, Myf5, MyoD, Mrf4, and myogenin [52]. Mice knocked out for the Pax7 gene displayed both defective muscle formation during embryonary period and reduced muscular regeneration capacity [38, 76, 77]. Lepper et al. (2009) have performed experiments regarding the conditional expression of Pax3 and Pax7, which showed that those transcription factors are only essential during the early life, being not determinant for muscular recovery in adult life [46]. Nonetheless, more recent works have shown Pax7 to remain essential for muscle regeneration in adult life. Pax7-expressing cells have been demonstrated to be important to regulate the environment in a sense that they might regulate other cell types during the postinjury period [78]. Besides, studies by von Maltzahn et al. (2013) showed disagreement to the previous study [46] by determining Pax7 to be necessarily expressed to regulate and maintain the myogenic potential of SCs [79]. More studies showing the relevance of the expression of other regulatory proteins for self-renewal have already been performed, allowing also elucidating the roles of MyoD, Myf5, and myogenin in this regulation. Those findings have been reviewed this year by Motohashi and Asakura [52]. MyoD has a noteworthy role in controlling apoptosis in SCs through the regulation of microRNAs (miR-1 and miR-206), which target binding sites in the 3′UTR of the Pax3 transcript. MyoD expression downregulates Pax3 and the antiapoptotic proteins Bcl-2 and Bcl-*x*. Considering that MyoD is downregulated in quiescent SCs, it reveals its involvement in the maintenance of this cell niche, by suppressing apoptosis [80].

Recent research at the protein level has elucidated the role of the Fas-associated death domain (FADD) in the maintenance of self-renewal in SCs. Phosphorylation of FADD at a specific site is directly connected to the noncommitment of SCs. Cells which underwent asymmetrical mitosis have displayed different distribution of the phosphorylated and unphosphorylated protein, in a fashion that the cells in which the phosphorylated protein accumulates tend to exhibit a less committed behaviour in addition to a stem-cell-like marker profile [81]. The asymmetrical divisions happening in SCs are coordinated by the differential activation of p38a mitogen-activated protein kinase (p38a/MAPK). The daughter cell where p38a/MAPK is activated begins to be determined as a more committed cell, since this pathway upregulated MyoD expression, whereas the cells with no p38a/MAPK activation remain MyoD− and less differentiated, replenishing the SCs pool [82].

The signalling for activation seems to be varied and is triggered in both physiological and pathological conditions and, thus, relevant for recovering muscle injuries as well as for the very formation and hypertrophy of those tissues. The p38a/MAPK-dependent asymmetrical division is also relevant considering its role of generating cells with upregulated myogenic regulatory factors.* In vitro* studies have reported nitric oxide (NO) and hepatocyte growth factor (HGF) as being relevant for their activation [83, 84]. Chemotaxis and activation of SCs were later also related to angiotensin II [85]. Not surprisingly, the coordination of SCs activation is also tight to the inflammatory pathways, conferring those cells the ability to respond to a proinflammatory environment, common in various sorts of myopathies and other conditions, which require muscular regeneration. In 2012, Paulsen et al. demonstrated the SCs responsiveness to cyclooxygenases 1 and 2 (COX1 and COX 2) [86]. Tumour necrosis factor (TNF) interacts with the p38a/MAPK resulting in the repression of the Pax7 locus, so that SCs activation is induced [87]. More recently, further studies have been performed and confirmed the SCs response to a proinflammatory environment [86].

### 4.3. Myogenic Regulatory Genes

It may be noted that the fate taken by the SCs is tightly related to the appearance of the product of certain genes; the differential expression of Pax3, Pax7, MyoD, Myf5, and Mrf4 seems to be connected to the self-renewal or differentiation towards a state of lesser potency. Thus said, the alternative fate for the SCs to self-renewal is its stimulation to perform muscle recovery by differentiating into myoblasts. Regulator proteins not expressed in self-renewing SCs tend, intuitively, to be expressed in the ones undergoing the process of differentiating into myoblasts. Thus, the myogenic process is tightly bound to the upregulation of MyoD, Myf5, and Mrf4 [88–91]. The regulation of those myogenic regulatory factors was also linked to Pax7 and Pax3 function, as discussed for the maintenance of the potency of the cell population [91].

## 5. Possibility of Stem Cell-Based Therapy in DMD

DMD is a genetic-based condition in which the afflicted individual suffers extensive and progressive muscular injuries as a result of frame shift mutations in the dystrophin gene. The great size and exon numbers in this gene result in a range of possible phenotypes and differing severity of the condition depending on the patient [14–17]. Nevertheless, the DMD patients tend to suffer severe muscular damage leading to outcomes ranging from diminished muscle control to loss of the cardiac and pulmonary functions, culminating in death [14–17]. Considering the SCs indispensable participation in muscular recovery, they have been regarded as important candidates to mediate cell-based therapies for DMD.

Earlier in 1989, the injection of myoblasts in mice models of DMD could successfully convert the myofibers to a normal expression of dystrophin [92]. However more recent attempts to induce regeneration of DMD-related damage in human dystrophic patients by transplanting myoblasts* in vitro* expanded and differentiated from SCs did not prove to be very promising. The negative results have been reported to be due to the insufficient migration of the myoblasts, immune reaction against the non-self-myoblasts transplanted, and death of the myoblasts [93–95]. The issues related to the inefficiency of the transplantation began to be addressed by further experimentation in both mice and monkeys. The increase in number of myoblasts injected has proved to be coherent with the amount of regenerated fibers [96]. Application of radiation to the affected muscles in order to enhance the release of myogenic related factors has also increased the success rates of the transplantations [97–100]. These later experiments have reached considerable increase in the restoration of dystrophin; however, they are still not ideal, taking into consideration that they are still inferior when compared to the already existing therapeutical approaches [101]. The immunogenicity of the myoblasts transplanted has been sought to be overcome both by the advances in immunosuppressants [101] and by inducing immune tolerance [102]. Other attempts have been done through transfection of the dystrophin microgene or full gene with viral vectors in order to genetically modify myoblasts to be transplanted [103–105]. Nonetheless, the restricted feasibility of those techniques for a clinical trial, concerning efficacy, safety, and cost, must be mentioned.

Earlier reports have demonstrated the augmentation in number of SCs in muscular tissue of DMD patients as a response to the disease. This evidence endorses the initial ideas of the potentiality of these cell populations for the treatment of the disease [106]. However, the depletion of SCs due to a Notch signalling deficiency seems to be a physiopathological issue regarding the abnormal muscle regeneration in mice models of DMD. The Notch signalling pathway has been shown to be necessary in order to keep the quiescent state of SCs [107]. Notch-reporter DMD-model mice enabled the authors to elucidate the mechanism by which this depletion takes place, since the model mice expressed a diminished activation of the Notch signalling leading to lack of proliferation control [107–109]. Taking into consideration that the increased number of SCs in DMD muscle is followed by depletion of those cells, it is suggested that allogeneic SCs populations with greater regenerative capability may become useful for cell-based therapies [106–109]. This year, the SCs derived from the extraocular muscle have successfully been transplanted into dystrophic animal models, in which they demonstrated great efficiency in regenerating dystrophin deficiency [110].

## 6. Conclusion

In spite of the many limitations inherent to cellular therapies, the SCs have been regarded for long as promising in the recovery of DMD-related injuries and in promoting mitigation of the outcome of the disease. The hopes for using SCs as a therapeutic alternative have been encouraged by the sum of positive results of some of the experiments cited throughout the text. Considering the accumulated knowledge on the existence of different SCs populations, it seems plausible to consider the use of specific populations in the therapeutic of dystrophic diseases, regarding the differential restorative capabilities of each of those populations. Specific SCs have been showing different capacities of injured muscle recovering, fomenting the search of SCs populations with the ideal profile to be used therapeutically for DMD-related injuries.

## Figures and Tables

**Table 1:** Biomarkers expressed in most SCs.

Biomarker	Functions	References
Pax7	Regulates self-renewal in SCs; maintains of myogenic potential; prevents precocious differentiation; regulates the expression of Myf5; promotes de specification of the SCs	[38, 78, 79, 111–113]

Pax3	Regulates proliferation of the SCs in conjunction with Pax7; is involved in myogenic differentiation; regulates the expression of Myf5	[114–119]

Myogenic Regulatory factor 5 (Myf5)	Is involved in embryonary myogenesis; promotes differentiation of SCs	[49, 120–123]

Barx2	Regulates postnatal muscle growth and regeneration; promotes activation and differentiation of SCs; is involved in myoblast fusion	[124–127]

M-cadherin	Cell adhesion protein; involved in cell-to-cell signalization that promotes proliferation of SCs; involved but not essential to myoblast fusion to form myofibers	[128–132]

c-Met	Required for adult skeletal muscle regeneration due to its role in myoblast migration and fusion	[133–136]

*α*7-integrin	Laminin receptor in the SCs, involved in the formation of neuromuscular and myotendinous cell junctions	[137–142]

Cluster of differentiation (CD) 34	Promotes cellular motility of SCs due to its antiadhesive function; is involved in the maintenance of the quiescence of SCs	[143–145]

Syndecan-3	Implicated in muscle regeneration; involved in the control of proliferation of SCs; role in angiogenesis	[146–148]

Syndecan-4	Implicated in muscle regeneration; involved in activation and proliferation of SCs	[146, 147, 149, 150]

Chemokine receptor type 4 (CXCR4)	Receptor for the ligand alpha-chemokine stromal-derived factor 1 (SDF-1); the activated receptor induces chemotaxis, calcium influx and activating the mitogen-activated protein kinase (MAPK) and AKT serine-threonine kinase by phosphorylation; involved in the control of SCs development	[151–154]

Caveolin-1	Modulates SCs activation during muscle repair	[142, 155–157]

Calcitonin receptor	Related to the maintenance of the quiescence of SCs	[158, 159]

Lamins A and C	Nuclear envelope proteins, involved in the regulation of SCs differentiation	[160, 161]

Emerin	Involved in the signalization for SCs differentiation	[161, 162]

**Table 2:** Summarized protocol for isolation and culture of SCs on Matrigel.

Step	Procedure
Obtaining the whole cell suspension	
Muscle collection	Properly dissect an entire muscle from the euthanized animals and transfer to ice-cold PBS for washing
Muscle preparation	(i) Dissect the muscle under light microscope in order to remove connective tissue, blood vessels, nerve fibers, and adipose tissue (ii) Cut and mince tissue for enzymatic digestion (iii) Digest with 0.2% collagenase with 10% fetal bovine serum (FBS) in DMEM medium
Obtaining a single-cell Solution	(i) Triturate mixture in order to obtain a single cell solution in up to 50 mL of DMEM with 2% FBS (ii) Pass the cell suspension through a 70 *μ*m cell strainer
Cell counting and concentration	(i) Count cells on hemocytometer (ii) Perform successive centrifugations to attain cells in a 200 *μ*L suspension (iii) The usual number of cells obtained from one mouse is of 2 × 10^6^ (iv) Suspension is to be done in 2% FBS in DMEM

Isolation	
SCs antibodies	(i) 1 *μ*L of antibodies against CD31-PE, CD45-PE, integrin-*α*7, and Sca-1-PE should be added to the 200 *μ*L cell suspension (ii) Incubation for 30 minutes on ice (iii) Wash cells with 1 mL of 2% FBS in DMEM by centrifuging at 2000 rpm for 3 minutes at 4°C (iv) Resuspend cells in 200 *μ*L of the 2% FBS and DMEM solution
Magnetic beads addition	(i) Add 10 *μ*L of anti-PE magnetic beads (ii) Incubate on ice for 30 minutes
Cell washing and suspension on MACS buffer	(i) Wash the cells twice in 1 mL of MACS buffer (ii) Resuspend in 1 mL of MACS buffer
Cell suspension elution and collection	Proceed MACS protocol by eluting the cell suspension through the column in order to obtain only the cells marked by the SCs-related antibodies

Culture	
Resuspension on myoblast medium	Resuspend cells in medium containing (i) Hams F10 medium (ii) 20% FBS (iii) Basic fibroblast growth factor (bFGF)
Plating	Cells should be plated in Matrigel-coated 10 cm plates containing 8 mL of the appropriate medium
Maintenance	Medium should be changed every 2 days and passaging is to be done before reaching 50% confluence
